# Patients’ access to and acceptance of community-based hepatitis C testing and treatment in Myanmar: A mixed-method study

**DOI:** 10.1371/journal.pgph.0000902

**Published:** 2023-06-16

**Authors:** Win Lei Yee, Anna Bowring, Bridget Draper, Daniel O’Keefe, Hla Htay, Kyi Thar Myint, Hnin Wai Phyo Aung, Yu Yu Win, Yi Yi Sein, Mary Mary, Aung Lin, Alisa Pedrana, Margaret Hellard

**Affiliations:** 1 Burnet Institute, Yangon, Myanmar; 2 Burnet Institute, Melbourne, Australia; 3 School of Public Health and Preventive Medicine, Monash University, Melbourne, Australia; 4 Myanmar Liver Foundation, Yangon, Myanmar; 5 Department of Infectious Diseases, The Alfred Hospital, Melbourne, Australia; 6 Doherty Institute and School of Population and Global Health, University of Melbourne, Melbourne, Australia; Universitas Sebelas Maret Fakultas Kedokteran, INDONESIA

## Abstract

Hepatitis C (HCV) infection elimination in low- and middle-income countries requires decentralised HCV services to increase testing and linkage to care. The CT2 Study investigated patients’ views of access to and acceptance of two community-based HCV care models in Myanmar using a mixed-methods approach. Point-of-care HCV testing and general practitioner-initiated HCV treatment were provided at two community clinics in Yangon, Myanmar–the Burnet Institute’s (BI) clinic focused on people who inject drugs (PWID), and the Myanmar Liver Foundation’s (MLF) clinic focused on people with liver-related diseases. Study staff administered quantitative questionnaires to 633 participants receiving anti-HCV antibody testing. Purposive sampling was used to recruit 29 participants receiving direct-acting antiviral treatment for qualitative interviews. Among participants completing quantitative questionnaires, almost all reported the clinic location was convenient (447/463, 97%), waiting time was acceptable (455/463, 98%), and HCV antibody and RNA testing methods were acceptable (617/632, 98% and 592/605, 97% respectively). Nearly all participants were satisfied with their clinic’s services (444/463, 96%) and preferred same-day test results (589/632, 93%). BI clinic participants were more confident that they understood HCV antibody and RNA results; MLF clinic participants were more comfortable disclosing their risk behaviour to staff and had slightly higher satisfaction with the overall care, privacy and secure storage of their information. In qualitative interviews, participants reported that flexible appointment scheduling, short wait times and rapid return of results increased the clinic’s accessibility. The simplified point-of-care testing and treatment procedures and supportive healthcare providers contributed to participants’ acceptance of the HCV care model. This decentralised community-based HCV testing and treatment model was highly accessible and acceptable to CT2 participants. Prioritizing patient-centred care, rapid provision of results, flexible appointments and convenient clinic locations can promote accessible and acceptable services which may in turn help accelerate progress in reaching HCV elimination targets.

## Introduction

An estimated 58 million cases of hepatitis C virus (HCV) infection occurred in 2019 [1] with a higher disease burden in low-and middle-income countries (LMICs) [2–4]. Most people are unaware of their HCV status [5,6] and despite highly effective treatment, an estimated 500,000 people die each year from HCV-related complications [7]. Whilst in high-income countries HCV transmission is predominately through the sharing of contaminated injecting equipment among people who inject drugs (PWID) [8], in LMICs, epidemics are predominately driven by unsterile medical procedures in formal and informal health care settings and/or unsterile injecting drug use [9,10]. If the World Health Organization’s (WHO) goal of an 80% reduction in new HCV infections and 65% reduction in HCV-related mortality by 2030 is to be achieved, we need a substantial increase in diagnosis and treatment [11] and affordably priced drugs [6,12], particularly in LMICs where treatment uptake and retention in care remain low [13].

HCV is a major health issue in Myanmar, which has a mixed epidemic due to healthcare-associated risks and high prevalence among PWID. An estimated 1.4 million (2.65%) people in the general population were HCV-antibody positive in 2015 [14,15], with prevalence among PWID over 56% [16]. There is no national level data of RNA prevalence and the liver disease burden, but it is estimated that, with the 26% spontaneous clearance rate, approximately 1.1 million people in Myanmar are living with chronic HCV infection [17]. Access to HCV testing and treatment in both public and private sectors has several challenges. Though HCV antibody testing is widely available, access to viral load testing is challenging due to high costs (approximately US$ 25) and limited availability in state and regional reference laboratories and some private laboratories. Interferon-based regimens were used for HCV treatment for many years. However, following the development of direct acting antivirals (DAAs) which have fewer side-effects and high efficacy, the Myanmar National Simplified Treatment Guidelines for Hepatitis C Infection recommends the use of interferon-free oral DAAs; this allows for HCV treatment to be prescribed in a primary care setting [18]. HCV treatment in private clinics is prohibitively expensive for most people in Myanmar. Prior to 2017, there was no national program providing free-of-charge HCV treatment. In 2017, following availability of DAAs, the National Hepatitis Control Program (NHCP) launched a national testing and treatment program [19]. DAA treatments are now available in public hospitals through this national program, private clinics with out-of-pocket payments by patients, some local and international non-government organizations (NGO) and philanthropic services. Between 2017 and 2020, the national program provided testing and treatment at 13 public hospitals. However, the free-of-charge treatment program was small [20] with only approximately 2000 people treated in 2018 [17]. A public-private partnership (PPP) program was initiated at government hospitals in 2018, with patient co-payments commonly required for both viral load testing and DAA treatment [20]. An estimated 11,000 people received HCV treatment through free-of-charge and cost-sharing programs at public hospitals between 2017 and 2019 [20], but there is no publicly available data on the treatment in NGO and private sector. To achieve Myanmar’s HCV elimination targets of treating 50% of eligible individuals with chronic HCV by 2030, it is imperative to improve access to affordable diagnostics and treatment.

Globally, there is increasing recognition that simplified, decentralised models of HCV care, provided by non-specialist clinical staff, are highly effective [21–23] and cost-effective [17] in increasing access to and retention in care. Consistent with this, Myanmar’s National Simplified Treatment Guidelines support general practitioners (GPs) to treat HCV in the community [18]. However, while studies have shown the benefits of decentralisation in LMICs [24], implementation research is scarce. The Burnet Institute, in collaboration with Foundation of Innovative New Diagnostics (FIND), the Myanmar Liver Foundation and the NHCP, implemented the Community-based Hepatitis C Testing and Treatment (CT2) study to evaluate the effectiveness of decentralised HCV care in Myanmar. This paper explores patients’ experiences and perceptions of access to and acceptance of HCV testing and treatment provided at two community clinics via CT2, and compares the experiences of PWID and people with liver-related health concerns.

## Methods

### Ethics statement

The study obtained ethical approval from the Department of Medical Research Ethics Committee in Myanmar (Ethics/DMR/2018/172) and the Alfred Hospital Human Research Ethics Committee (#244/17) in Australia. The trial was registered at ClinicalTrials.gov (NCT03939013). All the participants provided written informed consent to participate in the study. The study methods were conducted in accordance with relevant guidelines and regulations.

### Study design and setting

The CT2 study was conducted at two community clinics in Yangon, Myanmar: the Burnet Institute’s (BI) PWID clinic, and the Myanmar Liver Foundation’s (MLF) clinic for people with liver-related diseases. Each clinic employed a GP, a nurse and a laboratory technician, in addition to a peer worker for needle and syringe distribution and patient recruitment at the BI clinic. Participants who tested HCV-antibody positive using a rapid diagnostic test (SD BIOLINE–Standard Diagnostics Inc, South Korea) progressed to HCV RNA testing using the point-of-care (PoC) GeneXpert HCV VL assay which has the run time of 105 minutes. All positive participants were assessed for treatment and if eligible, offered DAA treatment at no cost. The CT2 study methodology has also been reported previously [25,26].

### Recruitment

#### The CT2 study

The study flyers were distributed at the government’s methadone treatment centers in Yangon and through an outreach worker to advertise the availability of the services at the Burnet clinic to PWID; clients attending were then consecutively enrolled in the study. In MLF clinic, the medical officers invited the new patients who came for HCV screening and the follow-up patients with known HCV antibody status but naive for HCV RNA testing. The participant recruitment was from January 2019 until September 2019. Potential participants were explained about the purpose and procedure of the study by the study nurse at both clinics and the written informed consent was collected from those who agreed to participate. The clients attending the two study sites who met the eligibility criteria (aged over 18 years, able to provide written informed consent, no known co-infection with HIV, hepatitis B or tuberculosis, HCV RNA testing and treatment naive, no known kidney dysfunction, no known drug interaction with sofosbuvir/daclatasvir) were invited to participate in the CT2 study. Total 634 participants were recruited into the main study but one participant withdrew from the study after the screening test.

*Acceptability Component*. All CT2 Study participants were asked to complete patient-completed surveys; these surveys included questions on acceptability of the service. These surveys were either completed by the patient themselves if they had sufficient literacy levels, or through nurse administered survey on an electronic tablet.

In addition, a subset of participants who were RNA positive and initiated HCV treatment were invited to participate in a qualitative interview after receiving sustained virologic response (SVR) result, typically after 12 weeks post-treatment. Purposive sampling was used to identify participants with a mix of gender, age, residential locations, injecting behaviours and SVR achievement. Twenty-nine participants were recruited for the study’s qualitative arm after being informed of its purpose and procedures and providing written informed consent.

### Data collection

Both quantitative and qualitative data collection took place at the study clinics ensuring privacy and confidentiality of the participants. Participants completed quantitative questionnaires, including questions exploring the acceptability of service provision, on three occasions throughout the study; screening (behavioural questionnaire to all participants), post-HCV diagnosis (acceptability questionnaire to all participants after receiving the results of anti-HCV antibody test and/or RNA test) and SVR testing visits (a combined behavioural and acceptability questionnaire to the participants after receiving the result of HCV RNA test at 12-week or 24-week post-treatment) [26]. Data were collected and managed using Research Electronic Data Capture (REDCap, Vanderbilt University, Nashville, TN, USA), with data hosted on a secure server at the Burnet Institute [27,28]. A five-point Likert scale was used to explore participants’ acceptance of clinic procedures, including their confidence in testing and comfort of blood collection, and their access to the model, including the convenience of the clinic location, clinical appointments and communication with healthcare providers.

Qualitative interviews were conducted face-to-face from December 2019 to March 2020 using a semi-structured interview guide exploring participant demographics, understanding of HCV infection, testing and treatment experience, perceptions of the service and referral, and access to and acceptance of the service. Interview length ranged between 30 and 60 minutes. All interviews were digitally audio-recorded, cross-checked to ensure data completeness and accuracy, transcribed verbatim and translated into English. Recruitment for qualitative interviews continued until response saturation was reached.

### Outcomes

The primary quantitative outcome measures to determine the acceptability of the testing and treatment pathways were (i) the proportion of respondents who reported drawing venous blood for testing acceptable (ii) the proportion of respondents who reported HCV antibody and RNA testing acceptable (iii) the proportion of respondents who were satisfied with the testing and treatment process and overall HCV care. The accessibility was determined by (i) the proportion of respondents who reported the clinic location was convenient (ii) the proportion of respondents who reported the wait time was reasonable. The qualitative outcome measures are the view of PWID and the general population with liver-related health concerns on the HCV testing and treatment services provided at two community clinics.

### Data analysis

This paper used survey data about the acceptability of service provision collected during post-HCV diagnosis and SVR testing visits. In addition, we used demographic and clinical information collected by GPs in case report forms through the customized electronic database Open Medical Record system (OpenMRS). Quantitative data were analysed descriptively to report response frequencies using Stata 15 (College Station, TX: StataCorp LLC). Given a small proportion of participants reported strongly disagree, disagree or neutral, five-point Likert scale responses were collapsed into 3-point scales; questions on agreement were collapsed to disagree (strongly disagree/disagree/neither agree nor disagree), agree and strongly agree. Similarly, questions on acceptance were collapsed to unacceptable (very unacceptable, somewhat unacceptable, neither acceptable nor unacceptable), somewhat acceptable and very acceptable. Questions on satisfaction were collapsed to dissatisfied (very dissatisfied, dissatisfied, neither dissatisfied nor satisfied), somewhat satisfied and very satisfied. Chi-square and Fisher’s exact tests were used to compare the differences in the results between participants by clinic.

Qualitative data were analysed thematically using NVivo12 (QSR International Pty Ltd., 2018). Text excerpts were coded and grouped into categories then structured into themes. Analysis was guided by the accessibility and acceptability domains of Tanahashi’s framework for effective coverage of health services [29]. Accessibility was defined as the availability of services within a person’s reasonable reach, including physical access and affordability dimensions, and acceptability was defined as willingness to seek services [29–31].

## Results

### Participant sample

**Fig 1:**
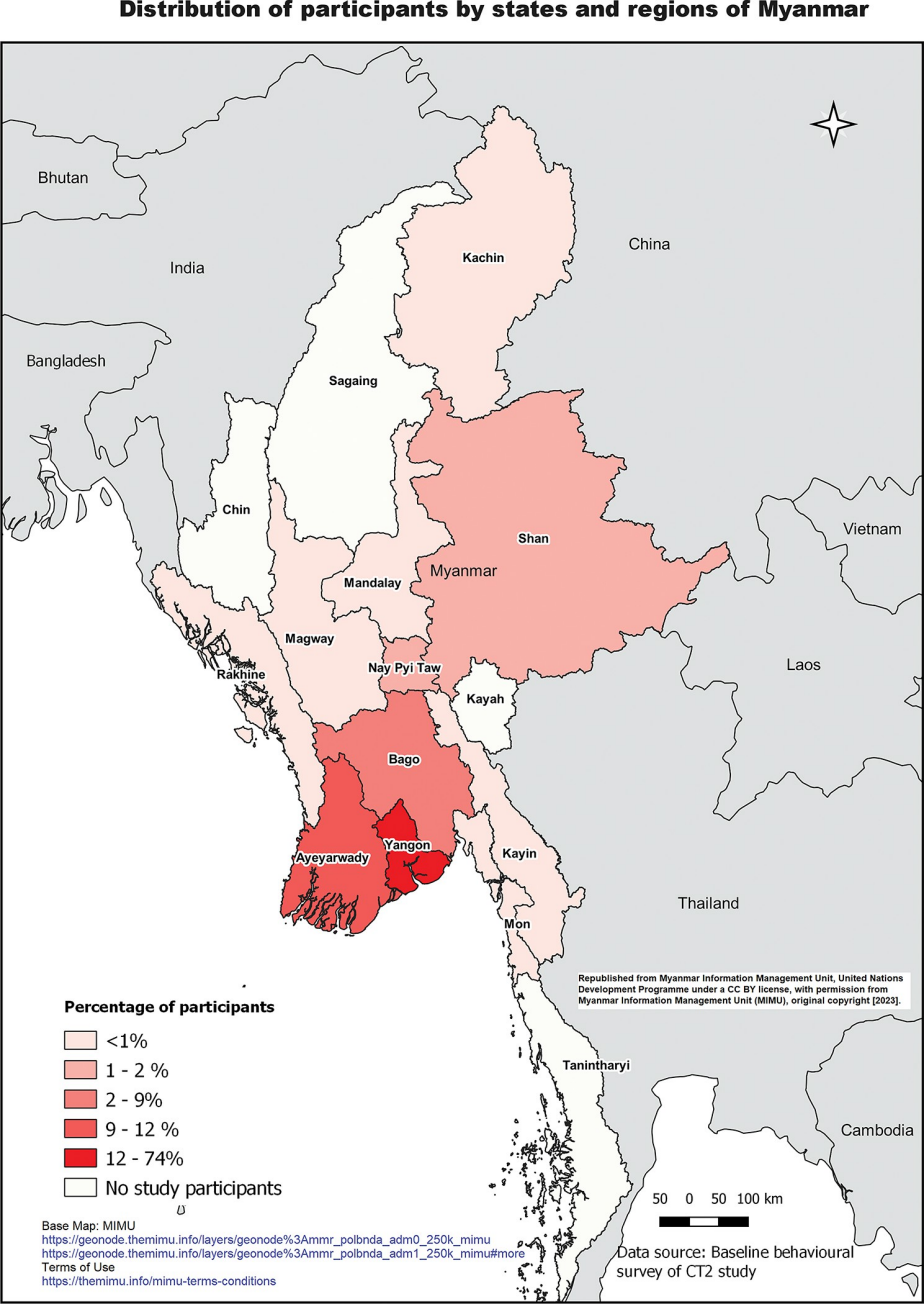
Distribution of survey participants by states and regions of Myanmar. Of participants (MLF n = 14, BI n = 15) who completed qualitative interviews, 20 (69%) were male and 9 (31%) were female, with median age 43 years (IQR 27–48). Twenty-three participants (MLF n = 8, BI n = 15) were residents of Yangon and 13 participants from the Burnet clinic were on methadone. Most participants (n = 26, 90%) achieved cure following HCV treatment.

The initial screening visit questionnaire was completed by all 633 participants, the post-diagnosis questionnaire by 632 (99%) participants, and the SVR questionnaire by 463 (73%) participants. Among 633 participants (MLF n = 380, BI n = 253), 405 (64%) were male and 228 (36%) were female. Median age was 42 years (IQR 31–53); 466 (74%) lived in Yangon (see Fig 1). Two hundred and sixty-five (42%) reported ever injecting drugs and 161 (25%) reported currently on methadone at screening (see Table 1). Four hundred and eighty-eight progressed to HCV treatment, of whom 463 (95%) completed SVR12 testing (456 completed the test within the defined time frame), with 427/463 (92%) achieving cure.

**Table 1:** Baseline demographic characteristics.

	Total N = 633 n (%)	MLF Clinic N = 380 n (%)	Burnet Institute N = 253 n (%)	Pearson’s chi-square test/ Wilcoxon rank sum test p-value
Sex, male	405 (64)	166 (44)	239 (94)	p<0.001
Median age, years (IQR)	42 (31–53)	50.5(39–59)	32 (27–40)	p<0.001
Residence location				p<0.001
Yangon	466 (74)	223 (59)	243 (96)	
Outside Yangon	167 (26)	157 (41)	10 (4)	
Currently prescribed methadone at screening	161 (25)	0 (0)	161 (64)	p<0.001
Previously tested for anti-HCV antibodies (self-report)				p<0.001
Never tested	91 (14)	12 (3)	79 (31)	
Tested previously	542 (86)	368 (97)	174 (69)	

### Quantitative data

Accessibility and acceptability responses of the participants are presented in Tables 2 and 3.

**Table 2:** Number and percentage of responses to SVR behavioural and acceptability survey.

	Total N = 463 N (%)	BI Clinic N = 162 N (%)	MLF Clinic N = 301 N (%)	Pearson’s chi-square test/ Fisher’s exact test p-value
The clinic location was convenient				
Strongly disagree/Disagree/Neutral	14 (3)	8 (5)	6 (2)	p<0.001
Agree	128 (27)	61 (38)	67 (22)	
Strongly agree	319 (69)	91 (56)	228 (76)	
Prefer not to answer	2 (1)	2 (1)	0	
Wait time was reasonable				
Strongly disagree/Disagree/Neutral	6 (1)	4 (2)	2 (1)	p<0.001
Agree	102 (22)	67 (41)	35 (11)	
Strongly agree	353 (76)	89 (55)	264 (88)	
Prefer not to answer	2 (1)	2 (1)	0	
Comfortable telling the clinic staff about risk behaviour				
Strongly disagree/Disagree/Neutral	4 (1)	4 (2)	0	p<0.001
Agree	66 (14)	52 (32)	14 (5)	
Strongly agree	390 (84)	103 (64)	287 (95)	
Prefer not to answer	3 (1)	3 (2)	0	
Secure storage of patient information				
Strongly disagree/Disagree/Neutral	1 (1)	1 (1)	0	p<0.001
Agree	64 (13)	51 (31)	13 (4)	
Strongly agree	397 (85)	109 (67)	288 (96)	
Prefer not to answer	1 (1)	1 (1)	0	
Privacy being respected				
Strongly disagree/Disagree/Neutral	4 (1)	4 (2)	0	p<0.001
Agree	63 (13)	48 (29)	15 (5)	
Strongly agree	394 (85)	108 (67)	286 (95)	
Prefer not to answer	2 (1)	2 (1)	0	
The staff were friendly and helpful				
Strongly disagree/Disagree/Neutral	1 (1)	1 (1)	0	p<0.001
Agree	56 (12)	44 (27)	12 (4)	
Strongly agree	404 (87)	115 (71)	289 (96)	
Prefer not to answer	2 (1)	2 (1)	0	
Satisfaction with the explanation of the testing process				
Very dissatisfied/Somewhat dissatisfied/Neutral	20 (4)	18 (11)	2 (1)	p<0.001
Somewhat satisfied	45 (10)	34 (21)	11 (4)	
Very satisfied	394 (85)	106 (65)	288 (95)	
Prefer not to answer	4 (1)	4 (2)	0	
Satisfaction with the explanation of the treatment process				
Very dissatisfied/Somewhat dissatisfied/Neutral	20 (4)	19 (12)	1 (1)	p<0.001
Somewhat satisfied	39 (8)	30 (19)	9 (3)	
Very satisfied	400 (86)	109 (67)	291 (96)	
Prefer not to answer	4 (1)	4 (2)	0	
Satisfaction of the overall HCV care				
Very dissatisfied/Somewhat dissatisfied/Neutral	18 (4)	17 (11)	1 (1)	p<0.001
Somewhat satisfied	46 (10)	33 (20)	13 (4)	
Very satisfied	398 (86)	111 (69)	287 (95)	
Prefer not to answer	1 (1)	1 (1)	0	

**Table 3:** Number and percentage of responses to acceptability survey.

	**Total** **N = 632** **N (%)**	**BI Clinic** **N = 252** **N (%)**	**MLF Clinic** **N = 380** **N (%)**	**Pearson’s chi-square test/Fisher’s exact test** **p-value**
Mode of transport				
Private vehicle	37 (6)	25 (10)	12 (3)	p <0.001
Taxi	200 (32)	59 (23)	141 (37)	
Public transport	351 (56)	150 (60)	291 (53)	
Walking/Bicycle/Other	44 (7)	18 (7)	26 (7)	
Transport costs (Myanmar Kyat)^♠^				
0	50 (8)	18 (7)	32 (8)	p = 0.82
100–10000	548 (87)	220 (88)	328 (86)	
10000+	32 (5)	12 (5)	20 (5)	
Acceptability of drawing blood from vein for antibody test				
Very unacceptable/Somewhat unacceptable/Neutral	14 (2)	12 (5)	2 (1)	p<0.001
Somewhat acceptable	105 (17)	62 (25)	43 (11)	
Very acceptable	513 (81)	178 (71)	335 (88)	
Acceptability of HCV antibody test ^€^				
Very unacceptable/Somewhat unacceptable/Neutral	15 (2)	14 (6)	1 (1)	p<0.001
Somewhat acceptable	74 (12)	57 (23)	17 (4)	
Very acceptable	543 (86)	181 (72)	362 (95)	
Confidence in understanding HCV antibody result				
Very unsure/Somewhat unsure/ Neutral	105 (17)	14 (6)	91 (24)	p<0.001
Confident	328 (52)	122 (48)	206 (54)	
Very confident	199 (31)	116 (46)	83 (22)	
Preference for same-day results				
No	27 (4)	14 (6)	13 (3)	p = 0.16
Yes	589 (93)	229 (91)	360 (95)	
Unsure	16 (3)	9 (4)	7 (2)	
	**Total** * **N = 605** **N (%)**	**BI Clinic** **N = 237** **N (%)**	**MLF Clinic** **N = 368** **N (%)**	
Acceptability of HCV RNA test (One response was missing)				
Very unacceptable/Somewhat unacceptable/Neutral	12 (2)	12 (5)	1 (1)	p<0.001
Somewhat acceptable	57 (9)	42 (18)	15 (4)	
Very acceptable	535 (88)	183 (77)	352 (96)	
Acceptability of drawing blood from vein for RNA test				
Very unacceptable/Somewhat unacceptable/Neutral	11 (2)	11 (5)	0	p<0.001
Somewhat acceptable	105 (17)	67 (28)	38 (10)	
Very acceptable	489 (81)	159 (67)	330 (90)	
Confidence in understanding RNA result				
Very unsure/Somewhat unsure/ Neutral	64 (11)	6 (3)	58 (16)	p<0.001
Confident	292 (48)	98 (41)	194 (53)	
Very confident	249 (41)	133 (56)	116 (32)	
Receiving RNA result (One response was missing)				
Same day	391 (65)	31 (13)	360 (98)	p<0.001
Next booked appointment	213 (35)	205 (87)	8 (2)	

♠ Two responses were missing.

^€^ Question: Having now had a HCV antibody test, how acceptable is this test to you?

*Total participants who had RNA testing.

#### 1. Clinic accessibility

Twenty-seven percent (128/463) of the survey respondents agreed and 69% (319/463) strongly agreed the clinic location was convenient (Table 2). Compared with MLF Clinic, Burnet clinic had a higher proportion of respondents who used public transport (60%, 150/252 vs 53%, 291/380) and a lower number of those who used taxis (23%, 59/252 vs 37%, 141/380); the differences are statistically significant (p<0.001) (Table 3). Most (87%, 548/632) spent ≤10,000 Kyat (USD7) on transportation costs (Table 3). Ninety-eight percent (455/463) of respondents also reported that waiting times at the clinic were reasonable (Table 2).

Twelve percent (20/167) of participants living outside of Yangon paid transportation costs of over 10,000 Kyat, compared to 3% (12/463) from Yangon (p <0.001). The use of public transport was similar between participants from Yangon and those outside of Yangon (56% vs 54%) but a higher proportion of participants living in Yangon used private vehicle (8% vs 1%) (p = 0.004). There was no significant difference between the groups for convenience of clinic location (Table 4).

**Table 4:** Accessibility to the clinic.

	**Total (n,%)** **N = 463^#^**	**Outside Yangon** **n,%**	**Yangon** **n,%**	**Pearson Chi-square test/ Fisher’s exact test** **p-value**
Convenient clinic location				
Disagree	14 (3)	5 (4)	9 (3)	p = 0.19
Agree	128 (28)	28 (21)	100 (30)	
Strongly Agree	319 (69)	98 (75)	221 (67)	
Prefer not to answer	2 (1)	0 (0)	2(1)	
	**Total (n,%)** **N = 632** ^**ψ**^	**Outside Yangon** **n,%**	**Yangon** **n,%**	**Pearson Chi-square test/ Fisher’s exact test** **p-value**
Mode of transport				
Private vehicle	37 (6)	1 (1)	36 (8)	p = 0.004
Taxi	200 (32)	62 (37)	138 (30)	
Public Transport	351 (56)	91 (54)	260 (56)	
Walking/Bicycle/Other	44 (7)	13(8)	31 (6)	
Transport costs (Myanmar Kyat)*				
0	50 (8)	10 (6)	40 (9)	p<0.001
100–10000	548 (87)	137 (82)	411 (89)	
10000+	32 (5)	20 (12)	12 (3)	

^#^SVR behavioural and acceptability survey respondents.

^ψ^Screening visit behavioural survey respondents.

*two responses missing.

#### 2. Service acceptability

Most respondents reported HCV PoC antibody and RNA testing as very acceptable (86%, 543/632 and 88%, 535/604 respectively). Drawing venous blood specimen for testing was rated very acceptable by 81% of respondents. Nearly all (93%, 589/632) respondents reported a preference for same-day test results. Of those who completed RNA testing, 65% (391/604) received their test result on the same day. Though preference for same-day RNA test result did not differ significantly between Burnet and MLF clinics, a significantly higher proportion of MLF participants than Burnet’s participants waited and received the result on the same day (98% vs 13%, p < 0.001) (Table 3).

Among those completing treatment and reaching the SVR time point, eighty-five percent (394/463), 86% (400/463) and 86% (398/463) of the survey respondents reported very satisfied with the explanations provided by healthcare staff regarding testing, treatment processes and the overall care received respectively (Table 2). Fifty-two percent (328/632) and 48% (292/632) of the participants reported being confident and 31% (199/632) and 41% (249/632) reported very confident they understood the meaning of the antibody test result and RNA test results respectively (Table 3). Further, 84% (390/463) of the respondents (98%) reported feeling very comfortable with informing clinic staff about potential HCV transmission risk behaviours, 85% (397/463) strongly agreed that their medical information was stored securely, 85% (394/463) strongly agreed that the clinic rooms had sufficient privacy for the consultations, and 87% (404/463) strongly agreed that clinic staff were friendly and helpful (Table 2).

There were noticeable differences in responses from Burnet and MLF clinics. A higher proportion of participants from MLF responded “very satisfied” or “strongly agree”, regarding the explanation of the testing (MLF 95% vs BI 65%, p<0.001) and treatment process (96% vs 67%, p<0.001), overall care (95% vs 69%, p<0.001), friendliness of staff (96% vs 71%, p<0.001), privacy (95% vs 67%, p<0.001) and secure storage of their information (96% vs 67%, p<0.001) (Table 2). However, a higher proportion of Burnet participants reported confidence in understanding test results; 94% and 97% of Burnet participants reported being confident or very confident in their understanding of antibody results and RNA results respectively, versus 76% and 85% of MLF clinic respectively (Table 3). MLF participants were more comfortable disclosing their risk behaviours with clinic staff than Burnet participants (Table 2).

### Qualitative data

#### 1. Clinic accessibility

*Clinic location*. Most participants, including those living outside of Yangon, reported that the CT2 clinics were easily accessible and close to good transportation routes. However, some transportation issues were noted for both clinics, such as the walking distance from nearby bus stops, and that the MLF clinic was located on a one-way street requiring longer taxi journeys. Nonetheless, participants reported that transportation issues were outweighed by the benefits of the clinics.

“This location is good and convenient for us. If the clinic was opened at another location, and even if it was far, we would go there and take the treatment.” (BI15, male)

*Travel*. More participants living further from the clinic, particularly outside Yangon City, reported challenges with travel (including cost). Furthermore, pre-treatment consultations often necessitated overnight stays in Yangon with associated costs if the participant planned to start treatment the next day. However, subsequent follow-up consultations could usually be completed without overnight stays.

“As I live away from Yangon, I have some problems with staying in Yangon. But it is not too bad.” (MLF06, female)

*Waiting time*. Services were generally completed in an hour except during consultations requiring viral load testing (approximately two hours). Clinic staff gave priority to patients living outside Yangon, ensuring same-day return of results.

“It would be four-five minutes at most [to see the staff]. Whenever I come, they [clinic staff] are always here. I don’t have to wait long.” (BI04, male)

*Appointment scheduling*. Most participants reported that appointment arrangements were flexible and negotiable. Appointment reminders were perceived as useful. Some participants needed to take days off work for appointments, but this was not reported as problematic. PWID taking daily methadone at the methadone center, which was open in the morning and close to the Burnet clinic, considered it convenient to attend the clinic in the morning.

“I take methadone everyday so it is okay for me to come in the morning.” (BI02, female)

“The doctor first asked me whether I was okay with that date or not… I had decided to come to the clinic any day they asked… I think it would be okay to negotiate the date… .” (BI08, male)

*Affordability*. Free services made the clinic more accessible to participants. Some participants of both MLF and Burnet clinics reported previous reluctance to access treatment due to reports from peers of high treatment costs at both public and private health centres. Participants, particularly those living outside Yangon, valued the free-of-charge service of the clinic so much so that their travel challenges and costs were considered worthwhile.

“A friend told me about this free of charge program. He said, ‘Hepatitis C project by Burnet for PWID has started and it’s free.’ That’s the reason I came here.” (BI01, male)

“It cost me thirty to thirty-five thousand MMK (US$ 20–23) to rent a car to and from the clinic. The clinic has provided charity treatment, so we have to spend this much money [on transportation].” (MLF05, female)

#### 2. Acceptability of the services

*Attitudes towards testing and treatment*. Participants related their experiences of CT2 services to previous experiences of attempting to access HCV services through government hospitals. Participants preferred the quick return of blood results and the one-stop-shop service model compared to government hospitals, which were reported to have prolonged waiting times and potentially unnecessary appointments.

“It’s fine. I must say it [MLF clinic] is very good. It is different from other laboratories and a public hospital…I am more comfortable here.” (MLF01, male)

“It [the government service] didn’t go well. There were many processes. It didn’t have a one-stop service, unlike this clinic… In the end, I lost my patience and decided not to get treated there.” (BI10, male)

Participants were also satisfied with the blood draws performed by laboratory staff, whom they rated as skillful. However, some participants, especially PWID, required more than one attempt to achieve successful blood draw. One PWID participant suggested those performing phlebotomy should be prepared to take advice from patients, who often had better knowledge of their own veins.

“It was successful only after four attempts because they couldn’t find the veins in earlier attempts. Being hard to find the veins due to collapsed veins has become quite usual for me since 2017. It doesn’t bother me.” (BI01, male)

*Understanding of testing and treatment procedures*. Participants reported that clinic healthcare providers satisfactorily explained testing and treatment procedures. However, while some participants reported knowing the difference between HCV antibody and RNA testing, some had difficulty recalling the types of tests received, drugs regimens and their side effects.

“[The nurse] explained about the tests for hepatitis C and viral load and then hepatitis B infection… and then … I don’t remember… I followed what they told but I don’t know the rest… I forgot” (MLF07, female)

Very few participants experienced medication side effects. Most were aware of the importance of taking drugs as scheduled.

“Yes, they explained [the side effects] to me and wrote many of them in it [booklet], but I didn’t suffer any of them.” (BI09, male)

Participants understood that DAAs could cure their infection but had little to say on their actions and effectiveness. Some participants stated they could not judge the effectiveness of DAAs, but believed the advice of the healthcare providers.

“We don’t understand much about medicine, so we believe what they told us. We don’t have medical knowledge so if they told us that we are cured, we believe we are cured.” (BI03, male)

“I was told I will be cured if I take these drugs and the chance is about ninety-five per cent.” (MLF02, male)

*Willingness for treatment*. Many participants reported a strong desire to be cured of their HCV infection, giving reasons of concern about disease progression, persuasion of family members and knowledge that cure was possible. Financial constraints hindered access to care, so they were grateful for the CT2 program’s free testing and treatment. Many of the participants knew Burnet Institute and MLF before the CT2 study, and their positive perceptions of these organizations possibly influenced their decision to participate. Some of the MLF participants who were on a waitlist to receive no-cost testing and treatment through various clinical trials conducted at the service described eagerness for the CT2 project to start.

“I decided on my own to wait for the study. When they contacted me about the commencement of study, I was thinking this program had a better guarantee [in quality] than other places. I believed we would get cured when the treatment was completed so I took part in it.” (MLF10, male)

“Then they told me to wait and put me on the list. They gave me a ring after one or two months. I was so happy to hear that. That’s why I came here to receive treatment.” (MLF09, female)

Participants also reported willingness to meet requirements in order to access treatment and achieve HCV cure. Clinic appointments and testing/treatment procedures did not impose a heavy burden.

“No, I didn’t have any [difficulties]. Since they had been treating my disease, I had to come no matter what … Since I wanted to be cured of this disease, I’d have to go to any place where I could get the medications that can treat my disease. No matter how hard it is.” (BI13, male)

“I came here regularly despite having difficulties… I mean financial problems including transportation cost.” (MLF 06, female)

*Relationship with healthcare providers*. Participants reported that they believed the clinic would keep their personal information confidential (as promised).

“No, I don’t think the information would be leaked. I have read what was written in the [consent form] which said the information would be kept confidential. Anyway, the doctors and physician who are treating us have high integrity.” (MLF04, male)

“They keep the information confidential that someone doesn’t want others to know, and I think it is good … Once they couldn’t reach me on my phone while I was at the meditation center so, they called my sister-in-law as they were worried something had happened to me. My sister-in-law didn’t know the reason why they had called as they kept it a secret.” (MLF07, female)

Participants of both clinics reported that healthcare providers were friendly, warm and kind. Participants felt that they were treated without discrimination by healthcare providers and could trust the providers and the treatment. They also described the healthcare providers as approachable and communicative, and available for ad hoc conversations via telephone if patients needed to report medication side effects or discuss other health issues.

“The main thing is that I have trust [in the clinic]. I got tested and was told and treated in a kind and warm manner. These all lead me to believe in the treatment.” (MLF10, male)

“If you ask me or other patients, you would get the same answer. All are warm and friendly and besides, they treated our infection for free. Even if we had to pay for the same treatment in other private clinics, we cannot get the same client relationship.” (BI09, male)

## Discussion

The study findings show a high level of acceptance of the decentralised CT2 HCV testing and treatment model. The findings are consistent with the overall outcome of the CT2 study, which demonstrated high retention in care (98% completed DAA therapy) and high cure rate (92% achieved SVR12) at both clinics [25].

Participants reported high satisfaction with the quality of HCV testing procedures and treatment. Wait times and the appointment system were reported as convenient and acceptable, and the time between HCV antibody and RNA testing and receiving results as highly acceptable. This finding contrasts with that of work in Australia on integration of PoC GeneXpert testing at harm reduction sites, which found that wait times of 105 minutes for RNA diagnosis were unacceptable to clients [32] and that 60 minutes would be preferrable [33]. The health system in Myanmar is constrained by poor infrastructure and a low doctor/patient ratio [34,35], causing patient congestion, and long wait times. Also, multiple appointments at hospital outpatient departments and laboratories are required to complete the treatment cascade. Procedures for laboratory tests are also complicated, often requiring out-of-pocket expenses, with viral load test mostly available only at the reference laboratory, with a long turnaround time. In our study, a simplified clinical pathway and PoC HCV testing enabled short wait and turnaround times, maximizing patients’ satisfaction and linkage to care.

Clinic locations were accessible to most participants, but participants living outside Yangon had more travel constraints due to additional costs that led to higher proportion of MLF participants waited for the same day result in order to avoid frequent clinic attendance. The BI clinic’s location approximately 300 meters from the methadone treatment center was convenient for PWID receiving daily methadone doses, which also enabled them to visit the clinic the following day of the HCV testing to receive the result. There is evidence that integration of HCV services with harm reduction sites improves access and linkage to care [24,36]. Our study model–not fully integrated, but near a methadone center–demonstrates the importance of convenient clinic locations for engaging PWIDs and enhancing HCV testing and treatment uptake.

Confidence in understanding HCV antibody and RNA results and satisfaction with services in our study were high, with slight variation between clinics. Quantitative data showed BI participants were more confident in understanding HCV antibody and RNA results than MLF participants. This could likely be the result of many PWID participants on methadone having previous exposure with the health education on HCV infection and the HCV screening test at the methadone program. The qualitative interview participants of both clinics were satisfied with healthcare providers’ explanations, but some did not remember all that were explained to them. They were found to have limited knowledge of DAA treatment which indicates the importance of emphasizing DAA treatment awareness in health education of HCV infection in the future. MLF participants were more comfortable in disclosing their risk behaviour to healthcare providers and more satisfied with explanations of testing and treatment, overall care, behaviour of staff, privacy and secure storage of their information. Stigma and discrimination are perennial issues for PWID, who are often stigmatized for their injection status, which can lead to distrust and avoidance of healthcare [37]. Consequently, PWID with previous experience of stigma, discrimination and stereotyping might be reluctant to discuss their risk behaviour with healthcare providers.

Three interlinking factors contributed to high acceptability of the CT2 community-based model. First, participants were eager to be cured and, consequently, prioritized clinic attendance and adherence to their treatment regimen. Challenges such as travel distance and associated income loss were considered negligible relative to the benefits of free and simplified testing and treatment at the CT2 clinics. The literature suggests that simplifying medication dosage and packaging lifts adherence [38]. Similarly, the simplified short-course treatment regimen, PoC testing process and short wait time in our study likely increased acceptability and adherence.

Second, participants were grateful to receive free HCV care. Previous work on healthcare practitioners’ perspectives on management of viral hepatitis in Myanmar found that the cost of diagnostic testing and treatment was the most significant barrier to treatment access among patients [39]. Likewise, some of our participants reported being unable to receive confirmatory testing for HCV diagnosis at other clinics due to the cost of RNA testing. Therefore, the no-cost HCV care provision of CT2 reinforced positive attitudes towards the services delivered and increased access. These two factors covering motivation for accessing treatment and gratitude for no-cost treatment in a context of limited treatment availability likely influence overall acceptability of services; with reported acceptability of services likely higher than if patients were asked to pay for services.

Third, a good patient–healthcare provider relationship was crucial, with most participants reporting trusting clinic staff–an important indicator of acceptance [40]. Our model of care enabled continuity of care for patients [41,42], strengthening relationships with healthcare providers and fostering ongoing trust in the model. Other studies have found that stigma and discrimination deter patients from seeking HCV testing and care [43–45] and that positive relationships improved patient engagement [46]. CT2 staff were perceived as friendly, ethical, competent and attentive, and built trust with patients easily. Ongoing training of healthcare providers that encompasses communication skills and addresses stigma by discussing medical ethics and patient vulnerabilities may help healthcare staff to build trusting relationship with patients.

There are some limitations to this study. Nearly all PWID patients at the BI clinic were male (consistent with national PWID population surveillance reports [16]); therefore, our findings may not represent the perspectives of female PWID in Myanmar. The healthcare providers in CT2 received training on study procedures, ethics and working with key populations, so the findings may not be generalizable to other services and their patients. Finally, HCV services were provided free of charge; further research on willingness to pay would be beneficial to inform scale-up implementation.

## Conclusion

Our findings indicated that short waiting time, flexible appointment scheduling, accessible clinic location and friendly healthcare providers trained in HCV treatment and stigma mitigation contributed to high access to and acceptance of the decentralised model of care. Free services and simplified testing and treatment procedures with quick turnaround times maximized patients’ adherence to treatment. This evidence of important service attributes is crucial for LMICs where testing and treatment need to be scaled up drastically to meet HCV elimination targets. Based on our findings, willingness and ability to pay are key factors when designing and delivering HCV services in LMICs. Incorporating these service attributes and considering affordability in future implementation of HCV services will promote testing and treatment uptake and retention in HCV care.

## Supporting information

S1 Text Qualitative interview guide for patients. (DOCX)

S2 Text Baseline behavioural survey. (DOCX)

S3 Text SVR12 behavioural and acceptability survey. (DOCX)

S4 Text Post-HCV diagnosis acceptability survey. (DOCX)
