# Supplementary material for: Patients’ access to and acceptance of community-based hepatitis C testing and treatment in Myanmar: A mixed-method study
Source: PLOS Glob Public Health. 2023 Jun 16;3(6):e0000902. doi: 10.1371/journal.pgph.0000902 (PMC10275420; doi:10.1371/journal.pgph.0000902)
Supplement: S1 Text — (DOCX) [file pgph.0000902.s001.docx]

**Interview Guide for Patients**

**Perceptions of Patients**

1. Demographic characteristics
2. Completed age
3. Gender
4. Township of residence
5. Estimated years of hepatitis C infection
6. Any previous treatment
7. Understanding of Hepatitis C infection
   1. How does the participant believe they became infected with HCV infection? Family history of HCV infection and treatment history?
   2. Understanding about HCV transmission risks
   3. Previous attempt to access treatment
   4. Any stigma and discrimination encounter when people know about their infection?
   5. What prompted the patient to come to the clinic for HCV testing?
   6. How did they know about this project? How were they involved in the study? Why?
8. Experience of testing and treatment
   1. Testing
      1. Confidentiality, privacy and informed consent
      2. Was the clinic location accessible to the participant?
      3. Blood Collection
      4. Waiting time
      5. Receiving the result – What do they think of the point-of-care GeneXpert test compared to the standard tests? Do they think the result is accurate?
      6. Receiving information about receiving treatment
      7. Understanding about testing, treatment, outcome and liver health
   2. Treatment
      1. How long was the time between diagnosis and treatment initiation?
      2. How was the process of initiating treatment?
      3. What are the challenges to come to the clinic to get testing (baseline and SVR12)? Challenges for regular visit to get DAA drugs?
      4. Were there any side effects that participant experienced?
      5. What do they know about the DAA drugs and their effectiveness
      6. Adherence - Any missed doses/ visits? What caused these missed doses/visits?
9. Perception of services provided
   1. Provider-patient communication (health education/ counselling)
      1. About hepatitis C (patient education)
      2. Drugs they are taking
      3. Tests/procedures done
      4. Prevention of re-infection
      5. Longer term monitoring
      6. Stigma/discrimination imposed by health care providers?
   2. Quality of the services
      1. What do they think of the communication of the staff at the clinic?
      2. What do they think of the clinic appointments? (Were there too many appointments? Did the participant feel the appointments were made at times/days acceptable to them? Did the participant feel they had input into the appointment times/days?)
      3. What do they think of the tests they underwent? (Were they all well explained and conducted in an acceptable way? Did the participant feel the tests were all necessary?)
      4. What do they want to suggest to improve the services at the clinic?
10. Patient perspectives on acceptability/accessibility
    1. Acceptability
       1. Testing and result return process
       2. Treatment initiation
       3. Treatment with DAAs
       4. Treatment at Community-based clinic setting vs hospital-based program
    2. Accessibility
11. If the participant was on a waiting list for treatment, how long have they been waiting?
12. How did they hear about study, why did they decide to get tested and have treatment?
13. Are you aware of other locations to receive HCV treatment? If so, why didn’t you go to one of these locations?
14. Perspective on being cured
15. Those who existed from the study – where were they referred? What further health services they sought?
16. Changes in behaviours after knowing the HCV infection status

i. Alcohol

ii. Injecting/ sharing needles

iii. Sexual behaviour

iv. Diet.

1. Patients experience of referrals

i. From where was the participant referred and to where?

ii.. How was it – waiting time, tests, treatment, consultation, follow-up, costs?

iii. What barriers to attending the clinic?

iv. What type of health practitioner and where would the patients preferred to go for treatment?
