# Supplementary material for: Patients’ access to and acceptance of community-based hepatitis C testing and treatment in Myanmar: A mixed-method study
Source: PLOS Glob Public Health. 2023 Jun 16;3(6):e0000902. doi: 10.1371/journal.pgph.0000902 (PMC10275420; doi:10.1371/journal.pgph.0000902)
Supplement: S2 Text — (DOCX) [file pgph.0000902.s002.docx]

| **PART A: IDENTIFIERS**  Part A is to be completed by a clinic staff member (e.g. clinician, receptionist). Please ensure each item is filled out correctly before handing to client to complete the behavioural survey starting from Part B. | | |
| --- | --- | --- |
| Clinic name:  Burnet Institute  MLF (Yangon)  MLF (Mandalay) | Clinician initials  e.g. first letter of each word of name | Visit date  //  (dd/mm/yyyy) |
| Client initials  e.g. first letter of each word of name | Client year of birth | Study ID |
| **PART B: PERSONAL DETAILS** | | |
| Please put a cross (X) in the box next to the response option you would like to choose. | | |
| Is the survey completed by the participants or with assistance from CT2 Study Staff member?   - Participant (by themselves) - With assistance from CT2 Study Staff member | | |
| 1. What sex were you assigned at birth? *(Please choose one)* | | |
| - Male | | |
| - Female | | |
| - Others | | |
| 1. What is your gender identity? *(Please choose one)* | | |
| - Male | | |
| - Female | | |
| 1. Which ward do you currently reside/stay/live in? | | |
| Ward: **__________________________** | | |
| 1. What is your highest attained level of education? *(Please choose one)* | | |
| - Illiterate | | |
| - Read and write | | |
| - Primary school | | |
| - Middle school | | |
| - High school | | |
| - College/University student | | |
| - Graduate/post-graduate | | |
| 1. Are you employed at the moment? | | |
| - Unemployed | | |
| - Employed | | |
| - Student | | |
| - Self-employed | | |
| - Retired | | |
| - Prefer not to answer | | |
| 1. What was your income in the past week? | | |

| _______________________MMK |
| --- |
| 1. Are you currently in a relationship? |
| - Married |
| - Separated |
| - Single |
| - Widowed |
| - Regular/de-facto partner (not married) |
| - Prefer not to answer |
| 1. What type of accommodation do you currently live in? |
| - Own residence |
| - Rented residence |
| - Staying at a parent’s house |
| - Staying at a friend’s house |
| - Homeless |
| - Shelter |
| - Prefer not to answer |
| **PART C: HEALTHCARE UTILISATION** |
| 1. Have you ever: *(Please check all that apply)* |
| - Been admitted to hospital |
| - Had a **blood** transfusion (e.g. get given blood over a few minutes or more) |
| - Had surgery |
| - Had injections (including vaccinations, medications – NOT BLOOD) |
| - Had stitches |
| - Had an endoscopy (Medical investigation of your internal organs using a thin tube with a camera) |
| - Had intravenous transfusions (NOT BLOOD PRODUCTS; e.g. fluid replacement, medications, nutrition) |
| - Had a catheter |
| - Had gum treatment |
| - Had teeth extractions |
| - Given birth |
| - Had a caesarean section |
| - Injected drugs |
| - Had renal dialysis |
| - Had a tattoo/scarification |
| - Had sex with a HCV positive person |
| - Had a family member with chronic HCV infection |
| - Had a family member with liver disease |
| 1. Have you ever been to a: *(Please check all that apply)* |
| - Quack |
| - Traditional medicine clinic |
| - Public clinic |
| - Midwife, even if not pregnant/giving birth |
| - Maternal Child Health centre |
| - Private generalist doctor |
| - Private clinic |
| - INGO clinic |
| - Pharmacy |
| - Sub Rural Health Centre (RHC) |
| - Rural Health Centre (RHC) |
| - Station hospital |
| - Township hospital |
| - Divisional hospital |
| - Speciality hospital |
| - Other medical service: _________________________________ |

| PART D: ALCOHOL USE |
| --- |
| 1. In the past ONE month, how often did you have a drink containing alcohol? (Please choose one) |
| - 4 or more times a week |
| - 2 to 3 times a week |
| - 2 to 4 times in the month |
| - Once in the month |
| - Never (→ skip to PART E) |
| 2. In the past ONE month, how many drinks containing alcohol did you have on a typical day when you drank? (Please choose one) |
| - 1 or 2 |
| - 3 or 4 |
| - 5 or 6 |
| - 7, 8 or 9 |
| - 10 or more |
| 3. In the past ONE month, how often did you have more than 6 standard drinks on one occasion? (Please choose one) |
| - Daily or almost daily |
| - Once a week |
| - Once in the month |
| - Never |
| 4. Alcohol consumption: |
| - Never |
| - Social |
| - Habitual |
| PART E: INJECTING DRUG USE |
| 1. Have you ever injected drugs? |
| - No (-> skip to PART F) |
| - Yes |
| - I don’t wish to say |
| 2. How old were you were first started injecting drugs? |
|  |
| 3. In the past six months, have you injected drugs? |
| - No |
| - Yes (go to Q5) |
| - I don’t wish to say |
| 4. If not injected in past six months, when did you last inject drugs? |
| / |
| month/year |
| 5. In the past six months, which drug did you most commonly inject? (Please choose one) |
| - Heroin |
| - Methamphetamine (Speed/Ice) |
| - Opium |
| - Methadone |
| - Suboxone/Buprenorphine |
| - Cocaine |
| - Prescription opioids (morephine/oxycodone) |
| - Benzodiazepines |
| - Hallucinogens |
| - Other; please specify: ___________________ |
| - I don’t wish to say |

| Injecting partners |
| --- |
| 6. In the past six months, how many different people did you inject with? |
| I don’t wish to say |
| PART F: INCARCERATION HISTORY |
| 1. Have you ever been incarcerated in prison? (Please choose one) |
| - No (Skip to Part G: Sexual Behaviors) |
| - Yes |
| - I don’t wish to say |
| 2. If yes, how many times? |
| I don’t wish to say |
| 3. When were you last released? |
| 🗌🗌/🗌🗌/ 🗌🗌🗌🗌 I don’t wish to say |
| 4. How long was your last sentence? |
| years, months, days I don’t wish to say |
| PART G: SEXUAL BEHAVIOURS |
| 1. Have you ever had sex with: (Please check all that apply) |
| - Men |
| - Women |
| - Never had sex (→ skip to PART H) |
| - I don’t wish to say |
| 2. In the past six months, have you had penetrative anal sex with:(Please check all that apply) |
| - Men |
| - Women |
| - Have not had sex in the past six months |
| - I don’t wish to say |
| 3. In the past six months, have you had insertive anal sex with: (Please check all that apply) |
| (insertive anal sex = your penis inserted into the rectum of another person) |
| - Men |
| - Women |
| - Have not had insertive anal sex in the past six months |
| - Not applicable to me (female) |
| - I don’t wish to say |
| 4. In the past six months, have you had receptive anal sex with:(Please check all that apply) |
| (receptive anal sex = penis of another person inserted into your rectum) |
| - Men |
| - Women |
| - Have not had receptive anal sex in the past six months |
| - I don’t wish to say |
| 5. In the past six months, how many different partners have you had anal sex with? (Please choose one) |
| - None (→ skip to PART H) |
| - One |
| - 2-5 |
| - 6-10 |
| - 11-50 |
| - More than 50 |
| - I don’t wish to say |
| 6. In the past six months, how often have you used condoms when having anal sex with casual partners? (Please choose one) |
| - I haven’t had anal sex with casual partners in the past six months |
| - Always used a condom |
| - Usually used a condom (more than half the time) |
| - Sometimes used a condom (less than half the time) |
| - Never used a condom |
| - I don’t wish to say |
| 7. In the past six months, how often have you used condoms when having anal sex with regular partners? (Please choose one) |
| - I haven’t had anal sex with regular partners in the past six months (→ skip to PART H) |
| - Always used a condom |
| - Usually used a condom (more than half the time) |
| - Sometimes used a condom (less than half the time) |
| - Never used a condom |
| - I don’t wish to say |
| 8. Thinking about your most regular sex partner, do you know their HIV status? (Please choose one) |
| - Yes, HIV positive |
| - Yes, HIV negative |
| - I don’t know/they haven’t had a test |
| - I don’t wish to say |
| 9. Thinking about your most regular sex partner, do you know their hepatitis C status? (Please choose one) |
| - Yes, hepatitis C positive |
| - Yes, hepatitis C negative |
| - I don’t know/they haven’t had a test |
| - I don’t wish to say |

| **PART H: EXPERIENCES OF STIGMA/DISCRIMINATION** | | | | | | | |
| --- | --- | --- | --- | --- | --- | --- | --- |
| The next few questions are trying to find out about your experiences of stigma and discrimination in healthcare settings. | | | | | | | |
| 1. In the past 12 months, when you have visited health care services (e.g. to see a doctor or nurse), to what extent have you experienced any stigma or discrimination   (e.g. avoidance, pity, blame, shame, rejection, verbal abuse, bullying, been made to wait/refused services) in relation to your: | | | | | | | |
|  | Never | Rarely | Sometimes | Often | Always | Not applicable to me | Prefer not to answer |
| History of injecting drug use? |  |  |  |  |  |  |  |
| History of incarceration/time in prison? |  |  |  |  |  |  |  |
| Hepatitis C status? |  |  |  |  |  |  |  |

| 1. In the past 12 months, to what extent do you think the following health care workers   have treated you poorly or differently to others attending the service? | | | | | | | |
| --- | --- | --- | --- | --- | --- | --- | --- |
|  | Never | Rarely | Sometimes | Often | Always | Not applicable to me | Prefer not to answer |
| **Nurse** has treated me poorly or differently to other people at the service |  |  |  |  |  |  |  |
| **Generalist doctor** has treated me poorly or differently to other people at the service |  |  |  |  |  |  |  |
| **Specialist at a hospital** has treated me poorly or differently to other people at the service |  |  |  |  |  |  |  |
| **One last question!** | | | | | | | |
| **Why are you having a hepatitis C test today? *(Please check all that apply)*** | | | | | | | |
| - I’m concerned about a recent exposure (e.g. I recently shared injecting equipment) | | | | | | | |
| - Injecting partner told me they had hepatitis C | | | | | | | |
| - Sexual partner told me they had hepatitis C | | | | | | | |
| - My friend recently got cured of hepatitis C | | | | | | | |
| - Routine check-up, no symptoms | | | | | | | |
| - My doctor suggested it | | | | | | | |
| - The staff at the clinic invited me to participate in this study | | | | | | | |
| - I got referred from: ____________________________ | | | | | | | |
| - Other reason – please list why: _______________________________________________ | | | | | | | |
| Thank you for completing this survey – your responses are very valuable to us and we appreciate the time you have taken to complete this survey. Please give the survey back to clinic staff. | | | | | | | |
