# Supplementary material for: Patients’ access to and acceptance of community-based hepatitis C testing and treatment in Myanmar: A mixed-method study
Source: PLOS Glob Public Health. 2023 Jun 16;3(6):e0000902. doi: 10.1371/journal.pgph.0000902 (PMC10275420; doi:10.1371/journal.pgph.0000902)
Supplement: S3 Text — (DOCX) [file pgph.0000902.s003.docx]

| **PART A: IDENTIFIERS** | | |
| --- | --- | --- |
| Part A is to be completed by a clinic staff member (e.g. clinician, receptionist). Please ensure each item is filled out correctly before handing to client to complete the behavioural survey starting from Part B. | | |
| Clinic name:  Burnet Institute  MLF (Yangon)  MLF (Mandalay) | Clinician initials  e.g. first letter of each word of name | Visit date  //  (dd/mm/yyyy) |
| Client initials  e.g. first letter of each word of name | Client year of birth | Study ID |
| **PART B: HEALTHCARE UTILISATION** | | |
| Is the survey completed by the participants or with assistance from CT2 Study Staff member?   - Participant (by themselves) - With assistance from CT2 Study Staff member | | |
| 1. In the last ONE MONTH, have you: *(Please check all that apply)* | | |
| - Been admitted to hospital | | |
| - Had a **blood** transfusion (e.g. get given blood over a few minutes or more) | | |
| - Had surgery | | |
| - Had injections (including vaccinations, medications – NOT BLOOD) | | |
| - Had stitches | | |
| - Had an endoscopy (Medical investigation of your internal organs using a thin tube with a camera) | | |
| - Had intravenous transfusions (NOT BLOOD PRODUCTS; e.g. fluid replacement, medications, nutrition) | | |
| - Had a catheter | | |
| - Had gum treatment | | |
| - Had teeth extractions | | |
| - Given birth | | |
| - Had a caesarean section | | |
| - Injected drugs | | |
| - Had renal dialysis | | |
| - Had a tattoo/scarification | | |
| - Had sex with a HCV positive person | | |
| 1. In the last ONE MONTH, excluding the visits to this clinic for the CT2 Study, have you ever been to a: *(Please check all that apply)* | | |
| - Quack | | |
| - Traditional medicine clinic | | |
| - Public clinic | | |
| - Midwife, even if not pregnant/giving birth | | |
| - Maternal Child Health centre | | |
| - Private generalist doctor | | |
| - Private clinic | | |
| - INGO clinic | | |
| - Pharmacy | | |
| - Sub Rural Health Centre (RHC) | | |
| - Rural Health Centre (RHC) | | |
| - Station hospital | | |
| - Township hospital | | |
| - Divisional hospital | | |
| - Speciality hospital | | |
| - Other medical service: _________________________________ | | |
| 1. Do you receive regular injections or infusions or transfusions? *(Please check all that apply)* | | |
| - Yes – blood transfusions | | |
| - Yes – other transfusions/infusions | | |
| - Yes – regular injections | | |
| - No | | |
| **PART C: ALCOHOL USE** | | |
| 1. In the last month, how often did you have a drink containing alcohol? *(Please choose one)* | | |
| - 4 or more times a week | | |
| - 2-3 times a week | | |
| - 2-4 times in the month | | |
| - Once in the month | | |
| - Never (**→ skip to PART D: OTHER DRUG USE)** | | |
| 1. In the last month, how many drinks containing alcohol did you have on a typical day when you drank?   *(Please choose one)* | | |
| - 1 or 2 | | |
| - 3 or 4 | | |
| - 5 or 6 | | |
| - 7, 8 or 9 | | |
| - 10 or more | | |
| 1. In the last month, how often did you have more than 6 standard drinks on one occasion? *(Please choose one)* | | |
| - Daily or almost daily | | |
| - Once a week | | |
| - Once in the month | | |
| - Never | | |
| 1. Alcohol consumption: | | |
| - Never | | |
| - Social | | |
| - Habitual | | |
| **PART D: INJECTING DRUG USE** | | |
| 1. Injected drugs in the past six months? *(Please choose one)* | | |
| - Yes | | |
| - No (**→ skip to Opioid Substitution Therapy)** | | |
| - I don’t wish to say | | |
| 1. In the past SIX months, which drugs have you injected? *(Please check all that apply)* | | |
| - Heroin | | |
| - Methamphetamine (e.g. ice, speed) | | |
| - Methadone | | |
| - Suboxone | | |
| - Other opioids | | |
| - Benzodiazepines | | |
| - Other drugs; please specify: ____________________ | | |
| - I don’t wish to say | | |
| 1. In the past ONE month, how many times did you inject drugs? | | |
| I don’t wish to say | | |
| 1. Have you ever re-used someone else’s syringe after they’ve used it? | | |
| - Yes | | |
| - No | | |
| - I don’t wish to say | | |
| 1. In the past ONE month, have you re-used someone else’s syringe after they’ve used it? | | |
| - Yes | | |
| - No | | |
| - I don’t wish to say | | |
| **Injecting partners** | | |
| 1. In the past six months, how many different people did you inject with in the same place and same time? | | |
| I don’t wish to say | | |
| **PART E: TESTING & TREATMENT PROCESS ACCEPTABILITY** | | |
| Thinking back to when you started hepatitis C treatment: | | |
| 1. How long after your first appointment did you start treatment?   - <7 days - 1-2 weeks - 2-4 weeks - >4 weeks | | |
| 2. Are you confident that you understood your treatment plan before starting treatment?   - Very unsure - Somewhat unsure - Neither confident nor unsure - Somewhat confident - Very confident | | |
| 3. How often did you need to attend the clinic to pick up your treatment medication?   - Daily - Weekly - Every four weeks - Only once | | |
| 4. Did you need to pay anything to pick up the treatment medication?   - Yes - No | | |
| 5. Did you see the doctor when you picked up the treatment medication?   - Yes - No | | |

| How satisfied were you with: | | | | | | | | | | |
| --- | --- | --- | --- | --- | --- | --- | --- | --- | --- | --- |
|  | Very satisfied | | Somewhat satisfied | | Neither satisfied nor dissatisfied | | Somewhat dissatisfied | | Very dissatisfied | |
| How the HCV testing process was explained to you |  | |  | |  | |  | |  | |
| How the test samples were taken |  | |  | |  | |  | |  | |
| How the test results were explained to you |  | |  | |  | |  | |  | |
| How the treatment process was explained to you |  | |  | |  | |  | |  | |
| The medical and nursing support available to you whilst on treatment |  | |  | |  | |  | |  | |
| The overall hepatitis C care you have received |  | |  | |  | |  | |  | |
| Thinking about having your testing and treatment through a community-based clinic, instead of at a hospital, how much do you agree or disagree with the following statements? | | | | | | | | | | |
|  | | Strongly agree | | Agree | | Neither agree nor disagree | | Disagree | | Strongly disagree |
| I trust that the staff at the community clinic are well-trained and can provide quality healthcare for hepatitis C | |  | |  | |  | |  | |  |
| I am confident that the test results the clinic staff provided to me are correct | |  | |  | |  | |  | |  |
| I am confident that the treatment plan the clinic staff put me on was the best for me | |  | |  | |  | |  | |  |
| I had enough information given to me before starting treatment | |  | |  | |  | |  | |  |
| If I asked for specific information, the doctors gave it to me | |  | |  | |  | |  | |  |
| I felt comfortable telling the clinic staff about my behaviours that might put me at risk of hepatitis C | |  | |  | |  | |  | |  |
| I felt confident that my medical information was securely stored | |  | |  | |  | |  | |  |
| I felt confident that my privacy was respected | |  | |  | |  | |  | |  |
| Thinking about the community clinic you attended, how much do you agree or disagree with the following statements? | | | | | | | | | | |
|  | | Strongly agree | | Agree | | Neither agree nor disagree | | Disagree | | Strongly disagree |
| I found the location of the clinic was convenient to me | |  | |  | |  | |  | |  |
| The wait time at the clinic was reasonable | |  | |  | |  | |  | |  |
| There was no delay in starting treatment | |  | |  | |  | |  | |  |
| The clinic rooms were clean | |  | |  | |  | |  | |  |
| The clinic rooms were private enough for the consultations | |  | |  | |  | |  | |  |
| The staff were friendly and helpful | |  | |  | |  | |  | |  |
| I would recommend this clinic to my friends/family | |  | |  | |  | |  | |  |
| Are you confident that you understand what you need to do to make sure you don’t get hepatitis C again?   - Very unsure - Somewhat unsure - Neither confident nor unsure - Somewhat confident - Very confident | | | | | | | | | | |
| Are you confident that you understand how to look after your liver now?   - Very unsure - Somewhat unsure - Neither confident nor unsure - Somewhat confident - Very confident | | | | | | | | | | |
