# Supplementary material for: Patients’ access to and acceptance of community-based hepatitis C testing and treatment in Myanmar: A mixed-method study
Source: PLOS Glob Public Health. 2023 Jun 16;3(6):e0000902. doi: 10.1371/journal.pgph.0000902 (PMC10275420; doi:10.1371/journal.pgph.0000902)
Supplement: S4 Text — (DOCX) [file pgph.0000902.s004.docx]

| **PART A: IDENTIFIERS** | | |
| --- | --- | --- |
| Part A is to be completed by a clinic staff member (e.g. clinician, receptionist). Please ensure each item is filled out correctly before handing to client to complete the behavioural survey starting from Part B. | | |
| Clinic name  Burnet Institute  MLF (Yangon)  MLF (Mandalay) | Clinician initials  e.g. first letter of each word of name | Visit date  //  (dd/mm/yyyy) |
| Client initials  e.g. first letter of each word of name | Client year of birth | Study ID |
| **PART B: ACCEPTABILITY OF TESTING PROCESS** | | |
| Please put a cross (X) in the box next to the response option you would like to choose. | | |
| Is the survey completed by the participants or with assistance from CT2 Study Staff member?   - Participant (by themselves) - With assistance from CT2 Study Staff member | | |
| 1. How did you first come to attend this clinic for hepatitis C testing and/or treatment? | | |
| - I was already attending this clinic for healthcare | | |
| - My usual doctor told me about it because I’m at risk of hepatitis C | | |
| - My friends/family told me to come get tested here | | |
| - Other: ______________ | | |
| 1. How did you get to the clinic today? | | |
| - Private vehicle | | |
| - Taxi | | |
| - Bus | | |
| - Train | | |
| - Walking | | |
| - Bicycle | | |
| - Other (specify): _______________ | | |
| 1. How long did it take you to get to the clinic today? | | |
| - <1 hour | | |
| - 1-2 hours | | |
| - 3-4 hours | | |
| - 5-6 hours | | |
| - >6 hours (more than one day) | | |
| 1. How much did it cost you each way? | | |
| __ ___ ___ ___ MMK | | |
| 1. Did you need to pay for food or accommodation or time off work to attend appointments? | | |
| - No | | |
| - Yes | | |
| 1. Food: __ __ __ __ MMK (per occasion attending appointment) | | |
| 1. Accommodation: __ __ __ __ MMK (per occasion attending appointment) | | |
| 1. Time off work/income lost: __ __ __ __ MMK (per occasion attending appointment) | | |
| 1. Childcare: __ __ __ __ MMK (per occasion attending appointment) | | |
| 1. How comfortable were you telling the healthcare provider about any behaviours that may have put you at risk of hepatitis C? (e.g. sexual behaviours, injecting drug use) | | |
| - Very uncomfortable | | |
| - Uncomfortable | | |
| - Neither comfortable nor uncomfortable | | |
| - Comfortable | | |
| - Very comfortable | | |
| 1. How confident were you that you understand what hepatitis C is? | | |
| - Very unsure | | |
| - Somewhat unsure | | |
| - Neither confident nor unsure | | |
| - Somewhat confident | | |
| - Very confident | | |
| 1. What does it mean if you have a positive hepatitis C antibody test? | | |
| - You are infected with hepatitis C | | |
| - You are not infected with hepatitis C | | |
| - You have been infected with hepatitis C, but you don’t know if you are actively infected now | | |
| 1. What does it mean if you have a positive hepatitis C RNA test? | | |
| - You are infected with hepatitis C | | |
| - You are not infected with hepatitis C | | |
| - You have been infected with hepatitis C, but you don’t know if you are actively infected now | | |
| 1. What are the chances of cure, following hepatitis C treatment? | | |
| - - Hepatitis C cannot be cured | | |
| - - Next to none (<10%) | | |
| - - Not very common (<25%) | | |
| - - Reasonable (at least 50%) | | |
| - - Very good (>90%) | | |
| 1. Can an infection with the hepatitis C virus cause liver cancer? | | |
| - - Yes | | |
| - - No | | |
| **PART C: (ANTIBODY TEST) ACCEPTABILITY OF THE ANTIBODY TEST**  *(*asked of everyone who had an antibody test)* | | |
| 1. Having now had a HCV antibody test, how acceptable is this test to you? | | |
| - Very unacceptable | | |
| - Somewhat unacceptable | | |
| - Neither acceptable nor unacceptable | | |
| - Somewhat acceptable | | |
| - Very acceptable | | |
| 1. If you chose “somewhat unacceptable” or “very unacceptable”, why was this test unacceptable to you? | | |
| - I feel the results will not be accurate | | |
| - It was uncomfortable | | |
| - It was painful | | |
| - It took too much time | | |
| - Other: ______________________ | | |
| 1. How acceptable was taking blood from your vein for this test? | | |
| - Very unacceptable | | |
| - Somewhat unacceptable | | |
| - Neither acceptable nor unacceptable | | |
| - Somewhat acceptable | | |
| - Very acceptable | | |
| 1. Would you prefer to have blood taken from your vein or from your finger? | | |
| - Vein | | |
| - Finger | | |
| 1. How confident do you feel that the result is correct? | | |
| - Very unsure | | |
| - Somewhat unsure | | |
| - Neither confident nor unsure | | |
| - Somewhat confident | | |
| - Very confident | | |
| 1. How long did you wait to receive the test result? | | |
| - <10 minutes | | |
| - 10-20 minutes | | |
| - 21-60 minutes | | |
| - 1-2 hours | | |
| - Over 2 hours to one day - 2-7 days - >7 days | | |
| 1. How long would you prefer to wait to receive the test result? | | |
| - <10 minutes | | |
| - 10-20 minutes | | |
| - 21-60 minutes | | |
| - 1-2 hours | | |
| - Over 2 hours to one day - 2-7 days | | |
| - >7 days | | |
| 1. ~~Did you have to:~~ *~~(select all that apply)~~* | | |
| - ~~Take the sample anywhere?~~ | | |
| - ~~Pay for the test?~~ | | |
| - ~~Pick up the result from anywhere except the clinic?~~ | | |
| 1. How comfortable were you having the test performed by a nurse? (not at a laboratory/hospital) | | |
| - Very uncomfortable - Uncomfortable - Neither comfortable nor uncomfortable - Comfortable - Very comfortable | | |
| 1. How comfortable were you receiving your test result from a nurse? (not a doctor) | | |
| - Very uncomfortable | | |
| - Uncomfortable | | |
| - Neither comfortable nor uncomfortable | | |
| - Comfortable | | |
| - Very comfortable | | |
| 1. Would you recommend to a friend that they have this test? | | |
| - Strongly recommend **not** having this test | | |
| - Recommend **not** having this test | | |
| - Neither recommend having nor recommend **not** having this test | | |
| - Recommend having this test | | |
| - Strongly recommend having this test | | |
| **PART D: ACCEPTABILITY OF RNA TEST** *(*asked of everyone who had a RNA test)* | | |
| 1. How long did you wait between having the antibody test (first test) and the RNA test (second test)? | | |
| - - <10 minutes | | |
| - - 10-20 minutes | | |
| - - 21-60 minutes | | |
| - - 1-2 hours | | |
| - - Over 2 hours to one day | | |
| - - 2-7 days | | |
| - - >7 days | | |
| 1. Having now had a HCV RNA test, how acceptable is this test to you? | | |
| - Very unacceptable | | |
| - Somewhat unacceptable | | |
| - Neither acceptable nor unacceptable | | |
| - Somewhat acceptable | | |
| - Very acceptable | | |
| 1. If you chose “somewhat unacceptable” or “very unacceptable”, what is the main reason for this? | | |
| - I feel the results will not be accurate | | |
| - It was uncomfortable | | |
| - It was painful | | |
| - It took too much time | | |
| - Other: ______________________ | | |
| 1. How acceptable was taking blood from your vein for this test? | | |
| - Very unacceptable | | |
| - Somewhat unacceptable | | |
| - Neither acceptable nor unacceptable | | |
| - Somewhat acceptable | | |
| - Very acceptable | | |
| 1. Would you prefer to have blood taken from your vein or from your finger? | | |
| - Vein | | |
| - Finger | | |
| 1. How confident do you feel that the result is correct? | | |
| - Very unsure | | |
| - Somewhat unsure - Neither confident nor unsure - Somewhat confident - Very confident | | |
| 1. How confident are you that you understand your test result? | | |
| - Very unsure | | |
| - Somewhat unsure | | |
| - Neither confident nor unsure | | |
| - Somewhat confident - Very confident | | |
| 1. How long did you wait to receive the test result?    - <10 minutes    - 10-20 minutes    - 21-60 minutes    - 1-2 hours    - Over 2 hours to one day    - 2-7 days    - >7 days | | |
| 1. How long would you prefer to wait to receive the test result? | | |
| - <10 minutes | | |
| - 10-20 minutes | | |
| - 21-60 minutes | | |
| - 1-2 hours | | |
| - Over two hours to one day | | |
| - 2-7 days - >7 days | | |
| ~~9. Did you have to:~~ *~~(please select all that apply)~~*   - ~~Take the sample anywhere?~~ - ~~Pay for the test to be performed?~~ - ~~Pick up the result from anywhere except the clinic?~~ | | |
| 1. How comfortable were you having the test performed by a nurse at a community clinic? (not at a laboratory/hospital) | | |
| - Very uncomfortable | | |
| - Uncomfortable | | |
| - Neither comfortable nor uncomfortable | | |
| - Comfortable | | |
| - Very comfortable | | |
| 1. How comfortable were you receiving your test result at a community clinic? (not at a hospital) | | |
| - Very uncomfortable | | |
| - Uncomfortable | | |
| - Neither comfortable nor uncomfortable | | |
| - Comfortable | | |
| - Very comfortable | | |
| 1. Would you recommend to a friend that they have this test? | | |
| - Strongly recommend **not** having this test | | |
| - Recommend **not** having this test | | |
| - Neither recommend having nor recommend **not** having this test | | |
| - Recommend having this test | | |
| - Strongly recommend having this test | | |
| **PART E: TESTING PREFERENCES** *(*asked of everyone who had an antibody test)* | | |
| 1. Which method of testing for **hepatitis C antibodies** would you prefer? | | |
| - Rapid test using blood from the vein with result available in 20 minutes | | |
| - Rapid test using blood from your finger with the result available in 20 minutes | | |
| - Standard blood test using blood from the vein where the sample is sent to the laboratory with result available in 1 week | | |
| - Rapid test using saliva from mouth with result available in 20 minutes | | |
| 1. What is the main reason for why you would prefer this method of testing for hepatitis C antibodies? | | |
| - It is quick | | |
| - I know the results will be accurate | | |
| - It does not hurt | | |
| - The nurse usually has trouble taking my blood | | |
| - Other: _________________________ | | |
| 1. Which method of testing for **hepatitis C RNA** would you prefer? | | |
| - Blood from the vein used for rapid PCR testing with result available in 2 hours | | |
| - Blood from the finger for rapid PCR testing with the result available in 1 hour | | |
| - Standard blood test using blood from the vein where the sample is sent to the laboratory with result available in 1 week | | |
| 1. What is the main reason for why you would prefer this method of testing for hepatitis C RNA? | | |
| - It is quick | | |
| - I know the results will be accurate | | |
| - It does not hurt - The nurse usually has trouble taking my blood - Other: _________________________ | | |
| 1. Would you prefer to get your hepatitis C test results on the same day as getting tested? | | |
| - Yes | | |
| - No | | |
| - Unsure | | |
| 1. Would you prefer to have your test done at a hospital, laboratory or this clinic? | | |
| - Hospital | | |
| - Laboratory | | |
| - Community clinic | | |
